# Beyond RPE: The Perception of Exercise Under Normal and Ketotic Conditions

**DOI:** 10.3389/fphys.2019.00229

**Published:** 2019-03-19

**Authors:** Olivia K. Faull, David J. Dearlove, Kieran Clarke, Pete J. Cox

**Affiliations:** ^1^Department of Physiology, Anatomy and Genetics, University of Oxford, Oxford, United Kingdom; ^2^Nuffield Department of Clinical Neurosciences, University of Oxford, Oxford, United Kingdom

**Keywords:** exercise, perception, RPE, ketoacidosis, anxiety

## Abstract

**Aim:**

Subjective perceptions of exercising exertion are integral to maintaining homeostasis. Traditional methods have utilized scores of ‘rating of perceived exertion’ (RPE) to quantify these subjective perceptions, and here we aimed to test whether RPE may encompass identifiable localized perceptions from the lungs (breathlessness) and legs (leg discomfort), as well as their corresponding measures of anxiety. We utilized the intervention of ketoacidosis (via consumption of an exogenous ketone ester drink) to independently perturb exercise-related metabolites and humoral signals, thus allowing us to additionally identify the possible contributing physiological signals to each of these perceptions.

**Methods:**

Twelve trained volunteers underwent two incremental bicycle ergometer tests to exhaustion, following ingestion of either an exogenous ketone ester or a taste-matched placebo drink. Cardiorespiratory measures, blood samples and perceived exertion scales were taken throughout. Firstly, two-way repeated-measures ANOVAs were employed to identify the overall effects of ketoacidosis, followed by generalized linear mixed model regression to isolate physiological predictors contributing to each perception.

**Results:**

Rating of perceived exertion was found to contain contributions from localized perceptions of breathlessness and leg discomfort, with no measurable effect of ketoacidosis on overall exertion. Leg discomfort, anxiety of breathing and anxiety of leg discomfort were increased during ketoacidosis, and correspondingly contained pH within their prediction models. Anxiety of leg discomfort also encompassed additional humoral signals of blood glucose and ketone concentrations.

**Conclusion:**

These results indicate the presence of localized components of RPE in the form of breathlessness and leg discomfort. Furthermore, subjective perceptions of anxiety appear to result from a complex interplay of humoral signals, which may be evolutionarily important when monitoring exertion under times of metabolic stress, such as during starvation.

## Introduction

Internal bodily perception of exercising exertion is a vital component in the maintenance of homeostasis. More than simply the sum of our physiology, understanding exercising humans as a psychosomatic whole (Borg, 1982) may lead to valuable insights: from the capacity for physical performance, through to barriers to exercise within population health (Clark, 1999; Cohen-Mansfield et al., 2003). Until now, measuring the subjective perception of exercising exertion has often utilized subjective scores of ‘rating of perceived exertion’ (RPE), touted as a single indicator of the degree of physical strain (Borg, 1982). RPE is thought to integrate a multitude of ascending and descending signals between the brain and periphery, including feed-forward motor drive as well as neural and humoral feedback (Hampson et al., 2001; Tucker, 2009). However, teasing apart the independent physiological signals contributing to RPE, and possibly the perceptual sub-components of this measure of exertion remain as unsolved challenges.

Whilst RPE can be an informative tool to encompass overall exertion perception, most human experiences are much more complex and nuanced. If we are to better understand subjective perceptions associated with athletic performance, deeper understanding of the potential contributing components to RPE is required. At its simplest level, these components may constitute location of exertion, for example compartmentalized perceptions of the legs or lungs. In light of this, breathlessness and leg fatigue have also previously been quantified during incremental exercise (Borg et al., 2010; Faull et al., 2016), albeit with almost parallel reported growth functions to RPE (Borg et al., 2010). Secondly, beyond localization of exertion, an important (and often overlooked) aspect to our exercise perception is the ‘affective’ or emotional qualities (Carrieri-Kohlman et al., 1996, 2001, 2009; Faull et al., 2016) such as the anxiety related to a sensation. Due to the salience of anxiety as a symptom, it may be that these affective qualities are powerful contributors to our homeostatic moderation of exercise, in addition to the intensity of a perception. Therefore, whilst fully understanding the host of signals contributing to perceptions of RPE may be beyond our current sensitivity, the aim of this manuscript was to break down exercising perceptions into both location and symptom quality as a first tangible step in understanding how we monitor and modulate perceived exertion during exercise.

Bodily perceptions during exercise may also have an important evolutionary role. During times of famine and starvation we produce ketone bodies, which act as a fuel for the brain and periphery to supplement low carbohydrate stores (Cahill and Owen, 1968; Cahill, 1970, 2006). Additionally, ketones act as a widespread signaling molecule, increasing peripheral fat metabolism and sparing carbohydrate to prolong survival (Robinson and Williamson, 1980). However, ketone bodies are acidic, inducing a mild ketoacidosis even within the physiological limits of controlled ketosis (0.2–7 mM) (Robinson and Williamson, 1980). The perception of acidosis, and furthermore ketoacidosis, may be important in maintaining homeostasis and the trade-off between physical exertion to overcome starvation, and the concomitant physiological strain. Importantly, ketoacidosis can also be induced via consumption of exogenous means, and specific ketone ester drinks have been reported to rapidly invoke blood ketone concentrations of the active D-βHB isoform to levels comparable with several days of starvation (2–5 mM/L) (Clarke et al., 2012a,b; Cox et al., 2016; Shivva et al., 2016; Stubbs et al., 2017). Therefore, using exogenous ketoacidosis to perturb normal exercising metabolism and examining the corresponding perceptual changes, we may be better able to both understand the independent physiological components contributing to exercising exertion, and potentially reveal ketones themselves as possible *perceptual* signaling molecules within the brain.

In this paper, we aimed to understand the simplest formation of potential sub-components that may contribute to the perception of RPE, and the physiological signals that may drive them. To do this, we first investigated the contributions of breathlessness, anxiety of breathing, leg discomfort, and anxiety of leg discomfort toward perceptions of RPE during incremental exercise both under placebo and exogenous ketotic conditions. By employing this tool of exogenous ketosis, we were able to then both separate aspects of physiology that are often tightly linked within exercise, and examine whether the evolutionarily important ketone molecules themselves may act as a homeostatic signal when monitoring bodily sensations.

## Materials and Methods

### Participants

Twelve healthy, athletically trained subjects [nine males, three females; age (mean ± SEM) 28 ± 1.6 years; height 185 ± 3.33 cm; weight 78 ± 3.5 kg; VO_2 max_ 56.5 ± 3.9 mL/min/kg; W_max_ 382.3 ± 69.8 W] participated in this study. Ethical approval was granted by the Oxfordshire Clinical Research Ethics Committee, and all participants provided written, informed consent.

### Protocol

Participants completed two incremental exercise tests to exhaustion on an electronically braked bicycle ergometer (Ergoline, Germany), separated by at least 5 days. Following an overnight fast, participants consumed a drink containing a ketone ester [330 mg/kg body weight of *(R)*-3-hydroxybutyl *(R)*-3-hydroxybutyrate ketone ester] or a calorie-free, taste-matched control drink prior to exercise, in a blinded, randomized and counterbalanced order. The ketone ester drink contained transesterified ethyl *(R)*-3-hydroxybutyrate with *(R)*- 1,3-butanediol, and has previously been shown to be a safe and effective way of elevating blood ketone levels (Clarke et al., 2012a,b; Cox et al., 2016; Shivva et al., 2016; Stubbs et al., 2017). The session order was determined in a randomized, counterbalanced and single-blinded fashion.

For blood measures, participants were inserted with a 22-gauge retrograde indwelling catheter into the dorsal vein of the hand immediately upon arrival. The hand was gently heated prior to blood sampling at rest and during exercise for arterialized blood measures (Forster et al., 1972), and blood samples were drawn at baseline (prior to drink consumption), immediately prior to exercise, and at exercise intensities of 100, 200, 300 W and maximal intensity prior to exhaustion. For each blood measure, a 1 mL blood sample was first drawn for blood gas measurements, which were immediately analyzed using a benchtop blood gas analyzer (Radiometer, Denmark) for measures of blood gasses, hemoglobin content, oxygen saturation and blood metabolites. Calculations of arterial pH and ${\mathrm{HCO}}_{3}^{-}$ from these samples were made using custom MATLAB scripts (MathWorks, Inc., United States) incorporating measured and expected hemoglobin, measured partial pressure of carbon dioxide (PCO_2_) and measured oxygen saturations (see Supplementary Materials and Methods for equations), and corrected arterial pH values were then transformed into hydrogen concentrations ([H^+^]) for further analyses (Forster et al., 1972), using a log transformation in Microsoft Excel^®^.

A second 2 mL blood sample was drawn at each interval for analysis of blood hormone and metabolite concentrations, with D-βHB immediately assayed using a portable analyzer (Abbott Laboratories, Ltd., United Kingdom). Samples were stored on ice, centrifuged (3,600 rpm for 10 min), and subsequently stored at -25°C until further analysis. Glucose, non-esterified fatty acids (NEFAs) and lactate were subsequently assayed using a commercial automated bench-top analyzer (ABX Pentra, France), and insulin assays were performed using an ELISA kit (Mercodia, Sweden).

To collect respiratory gas measures, participants breathed through a facemask (Hans Rudolf, Kansas City, MO, United States) attached to a low-resistance turbine and gas flow analyzer (Cortex Metalyzer 3B, Cranlea Human Performance, Ltd., United Kingdom) to measure the breath-by-breath composition of respiratory gasses and ventilatory parameters (Metasoft Studio Software, Cortex, Version 3.9.9). Respiratory measures were averaged and recorded over approximately 2 min both prior to drink consumption and immediately before exercise. Heart rate was measured and recorded using a chest strap (Polar T31, Polar Electro, Inc., Lake Success, NY, United States) wirelessly connected to the Metasoft Studio.

Incremental exercise to exhaustion was conducted on an electronically braked bicycle ergometer (Ergoline, Germany) for the determination of maximal volume of oxygen consumption (VO_2 max_) and maximal work (W_max_) (Cortex Biophysik, Germany). At least one male and one female experimenter were present at both exercise sessions for each participant. Exercise began at 100 W, and increments of either 25 or 50 W were undertaken every 3 min until volitional fatigue. Respiratory measures were averaged and recorded over approximately 30 s within the last minute of each exercise step, and ∼5 mL blood samples were drawn at 100, 200, 300 W and maximal exercise. Subjective scores of RPE (“What is your perceived exertion?”), breathlessness (“How breathless are you?”), leg discomfort (“What is your leg discomfort?”), anxiety of breathing (“How anxious are you about your breathing?”) and anxiety of leg discomfort (“How anxious are you about your leg discomfort?”) were recorded in a randomized order on a linear scale of 0–10 (from “No [breathlessness/leg discomfort/anxiety of breathing/anxiety of leg discomfort]” = 0 to “Maximal [breathlessness/leg discomfort/anxiety of breathing/anxiety of leg discomfort]” = 10) both immediately prior to exercise and at the end of each incremental step (see Supplementary Material for rating scales).

### Statistical Analysis

Preliminary statistical analyses were performed using GraphPad Prism (version 7, GraphPad Software, Inc., San Diego, CA, United States). All variables were first analyzed using a two-way analysis of variance (ANOVA) of the intervention (ketone or control) and cycle ergometer power. A *p-*value of < 0.05 was taken to indicate statistical significance. Self-reported subjective variables (RPE, breathlessness, anxiety of breathing, leg discomfort and anxiety of leg discomfort) were then further explored using generalized linear mixed effects regression models in R (Faraway, 2017) (R version 3.4.1, RStudio version 1.0.143). Mixed-effects regression models allow accountability for repeated measures taken within subjects, such as across an experimental exercise test, without violating residual independence or autocorrelation assumptions of linear regression models.

Following tests for data normality (Supplementary Material), models were fitted to each of the psychological variables using a generalized linear mixed effects model (glmer; Bates et al., 2015), with maximum likelihood (laplace approximation) and a log-normal link function. A subject-specific random effect was included in each model, to allow a different model intercept to be fitted for each subject. Data points where all measured blood, heart rate and respiratory variables were present were used for data modeling, and all variables were centered and scaled prior to inclusion. Two subjects were excluded from modeling analyses due to missing heart rate data.

To firstly test the potential contribution of breathlessness, leg discomfort, anxiety of breathing, and anxiety of leg discomfort toward the perception of RPE, a global model was fitted including covariates for power, heart rate, sex (and their interactions), as well as the four measured psychological components and the interactions between power and both leg discomfort and breathlessness (see Supplementary Material for explanation of included variables). A parsimonious model of the key predictor variables was then identified using backward elimination of statistically non-significant terms (*p* > 0.05) until the model contained all significantly contributing variables. This final model was then formally compared to a null model (containing only covariates for power, heart rate, sex and their interactions) using the ANOVA function with type III Wald chisquare tests (R stats package), to assess improvement in fit to the data.

To then test which physiological factors may drive each of the four psychological components, initial global models were fitted containing all hypothesized contributing variables (power, heart rate, sex and their interactions, age, hydrogen ion concentration [H^+^], lactate ion concentration [Lactate^-^], D-βHB ion concentration [βHB^-^], glucose concentration [Glucose], ventilation (for breathlessness and anxiety of breathing only), and an interaction term between power and [H^+^] (power × [H^+^])) (see Supplementary Material for included variables). Parsimonious models of the key predictor variables were then identified using backward elimination of statistically non-significant terms (*p* > 0.05), until each model model contained all significantly contributing variables. Some non-significant variables were retained if their interaction with another variable was significant, to preserve a hierarchical, well-formulated model (Morrell et al., 1997). These reduced models were tested against the initial global models using the ANOVA function in R and type II (no interaction terms) or III (interaction terms present) Wald chisquare tests, to confirm that the reduced set of variables did not impair the quality of the model fit. A *p-*value of < 0.05 was taken to indicate statistical significance of an independent effect of each parameter, accounting for all other parameters in the model. Finally, full model R^2^ and fixed effect *R*^2^-values for each global and reduced model were calculated using the r2beta function within the r2glmm package, using the standardized generalized variance approach (Jaeger et al., 2016), and the predicted data was plotted against the measured data for visualization of model accuracy (Figure 4).

## Results

### Intervention Effects of Exogenous Ketosis

**FIGURE 1:**
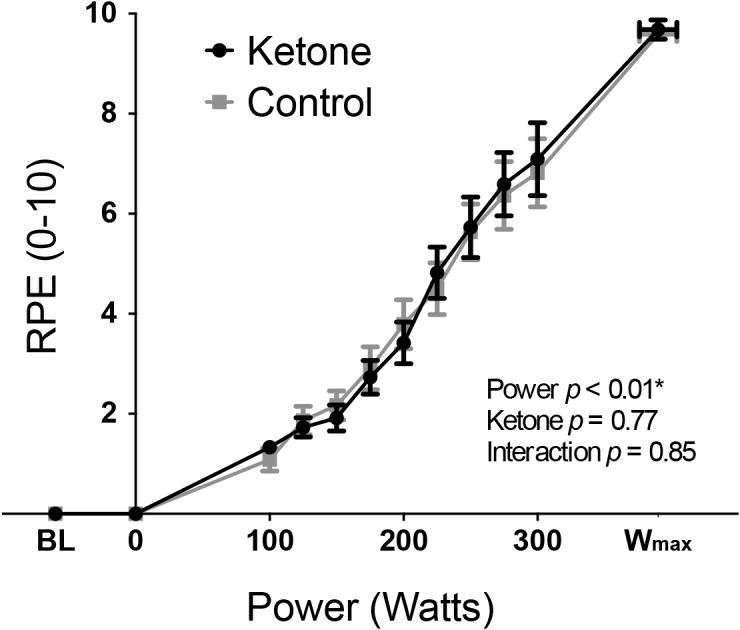
Rate of perceived exertion (RPE: mean ± SEM) plotted against power during incremental exercise under ketosis or placebo control conditions. *p*-values represent analysis of variance (ANOVA) results for power, ketone ester intervention and an interaction effect. Statistical significance (denoted by ^∗^) was taken at *p* < 0.05. BL = baseline.

**FIGURE 2:**
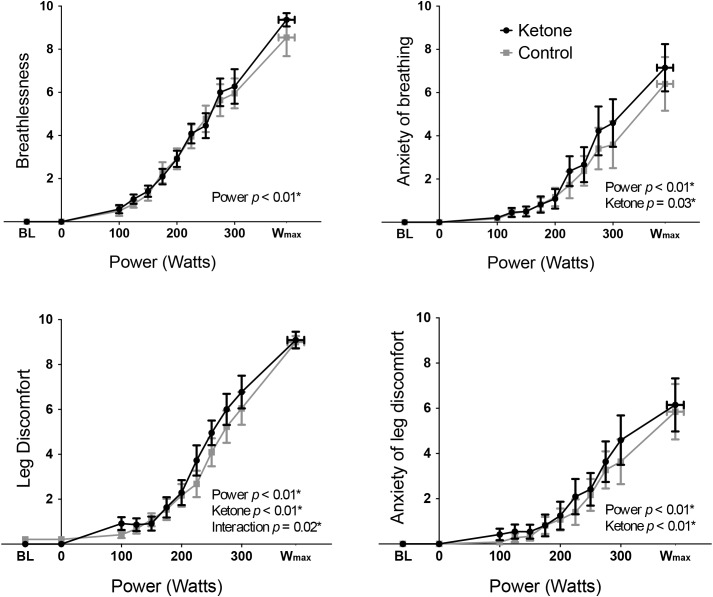
Mean ( ± SEM) perceptions of breathlessness, anxiety of breathing, leg discomfort, and anxiety of leg discomfort plotted against power during incremental exercise, under ketosis or placebo control conditions. *p*-values represent analysis of variance (ANOVA) results for power, ketone ester intervention, and interaction effects for each perception. ^∗^*p* < 0.05. BL = baseline.

Exercise performance (W_max_) was not significantly different between KE (mean ± SEM: 393 ± 22 W) and control (389 ± 20 W) conditions (*T* = 1.07, *p* = 0.31). Overnight fasted D-βHB levels were 0.2 ± 0.0 mM/L in both conditions at baseline, while D-βHB increased significantly to 3.7 ± 0.3 mM/L following KE consumption prior to exercise. The main effect of a KE on all other physiological variables are presented elsewhere (Dearlove et al., in press). To address the aims of this manuscript, the significance of the contributions of both the KE intervention and cycle ergometer power (both fixed effect measures, determined by the study protocol design) toward the measured perceptual quantities were firstly assessed via two-way repeated measures ANOVA analyses. For both RPE (Figure 1) and breathlessness (Figure 2), power was the only significantly contributing variable, while leg discomfort demonstrated a significant effect of power, KE intervention and an interaction effect (Figure 2). Anxieties of breathing and leg discomfort both showed a significant effect of both power and KE intervention (Figure 2), with no interaction effect. Full ANOVA results are provided in the Supplementary Material.

### RPE Model

**FIGURE 3:**
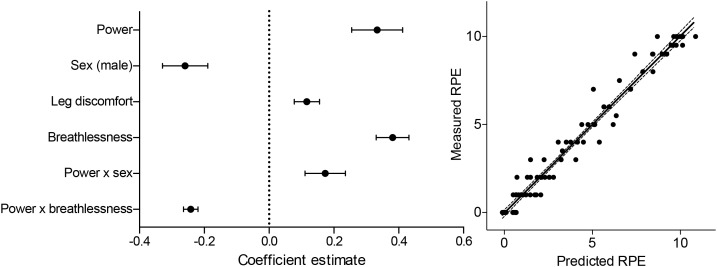
Summary of the model coefficients (left) and predicted vs. measured outcomes for the generalized linear mixed effects regression model (glmer) of RPE (RPE). Glmer was fitted using a log-normal link function to a gaussian distribution. All predictor variables were centered and standardized before inclusion into the model, and are represented as mean estimates ( ± standard deviations).

The parsimonious generalized linear mixed effects RPE model contained significant contributions from both breathlessness and leg discomfort, but neither anxiety score (Figure 3). In addition, a negative interaction between power and breathlessness was also retained within this model, indicating that the increases in breathlessness with power ‘tail off’ toward maximal exertion. The modeling of RPE scores was improved in comparison to the null model, which contained only physiological variables [ANOVA comparison: Null model: *R*^2^ = 0.96, Residual Degrees of freedom (RDoF) = 80, Akaike’s Information Criterion (AIC) = 254.1, Bayesian Information Criterion (BIC) = 276.5; vs. Final model: *R*^2^ = 0.97, RDoF = 80, AIC = 195.2, BIC = 217.6; Comparison Chi-squared = 58.9, *p* < 0.001]. Positive, significant effects of both breathlessness (*T* = 7.5, *p* < 0.001) and leg discomfort (*T* = 3.0, *p* < 0.001) were found to contribute to RPE score, explaining 31 and 8% of the RPE variance respectively (see Supplementary Table 2 for full results). A negative effect of the interaction between power and breathlessness (*T* = -10.7, *p* < 0.001) was also observed, explaining 58% of the RPE variance. No significant, independent contribution of anxiety of either breathing or leg discomfort toward RPE score was demonstrated.

### Compartmentalized Perceptual Models

#### Breathlessness

**FIGURE 4:**
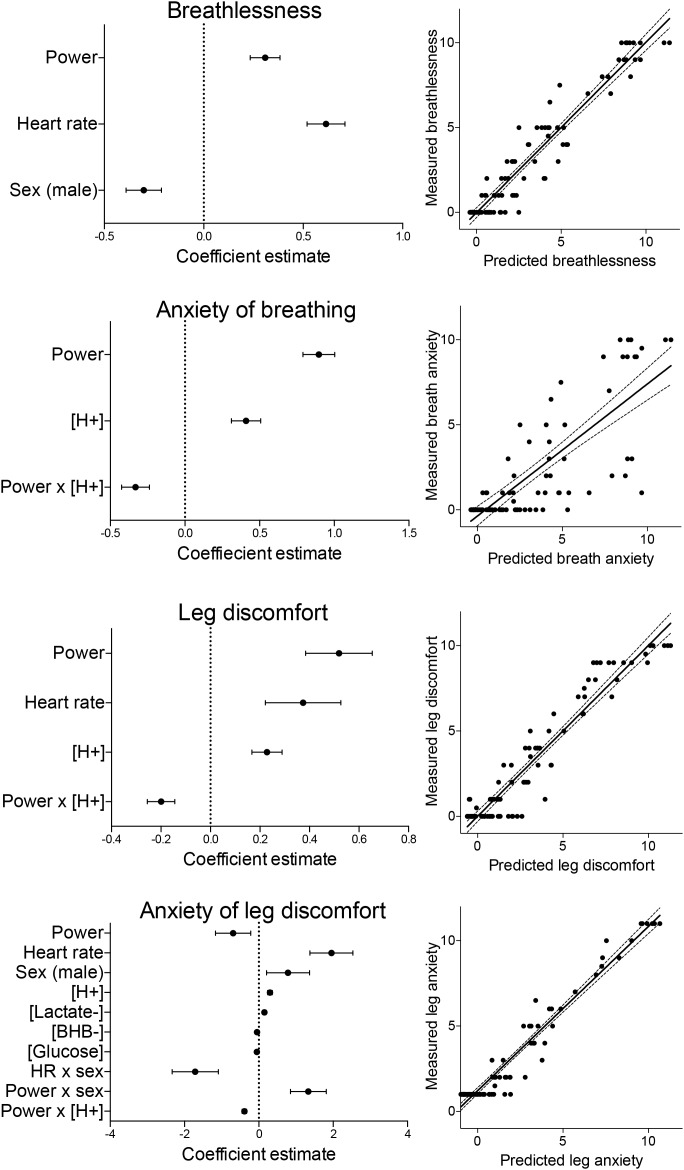
Summary of the model coefficients (left) and predicted vs. measured outcomes for the generalized linear mixed effects regression models (glmer). Models were fitted for ratings of breathlessness, anxiety of breathing, leg discomfort and anxiety of leg discomfort, and each glmer was fitted using a lognormal link function to a gaussian distribution. All predictor variables were centered and standardized before inclusion into the model, and are represented as mean estimates ( ± standard deviations).

Results of the full model fit for breathlessness are presented in Table 1, demonstrating a significant, positive, independent effect of power, and negative effects of sex (male) and glucose (Table 1). The reduced, parsimonious model of breathlessness demonstrated significant, driving positive effects of power and heart rate, with a negative effect of sex (male), and a model *R*^2^-value of 0.93 (Figure 4 and Supplementary Table 3).

**Table 1:** Model summaries and coefficients for global model fits to each of the compartmentalized perceptions of breathlessness, leg discomfort, anxiety of breathing (breath anxiety), and anxiety of leg discomfort (leg anxiety).

	Breathlessness			Breath anxiety			Leg discomfort			Leg anxiety		
				
Fixed effects	*Coefficient*	*T*	*p*	*Coefficient*	*T*	*p*	*Coefficient*	*T*	*p*	*Coefficient*	*T*	*p*
Intercept	1.91	7.95	<0.01^*^	0.48	0.81	0.42	1.39	4.67	<0.01^*^	0.17	0.31	0.76
Power	1.10	2.95	<0.01^*^	0.36	0.43	0.67	0.73	1.65	0.10	-0.89	-1.83	0.07
Heart rate	0.09	0.32	0.75	0.22	0.27	0.79	0.48	1.08	0.28	2.13	3.61	<0.01^*^
Sex (male)	-0.36	-2.50	0.01^*^	-0.03	-0.08	0.94	0.03	0.11	0.92	0.88	1.51	0.13
[H^+^]	-0.01	-0.21	0.84	0.37	2.90	<0.01^*^	0.14	1.96	0.05^*^	0.30	4.66	<0.01^*^
[Lactate^-^]	0.07	1.05	0.30	-0.02	-0.19	0.85	0.03	0.49	0.63	0.23	3.41	<0.01^*^
Ventilation	-0.04	-0.39	0.70	0.34	1.53	0.13	-	-	-	-	-	-
[βHB^-^]	-0.04	-1.91	0.06	-0.04	-0.98	0.33	-0.01	-0.40	0.69	-0.04	-1.42	0.15
[Glucose]	-0.08	-2.08	0.04^*^	0.08	1.09	0.27	-0.04	-1.41	0.16	-0.05	-2.02	0.04^*^
Power × HR	-0.10	-0.96	0.34	-0.16	-0.62	0.53	-0.03	-0.28	0.78	-0.24	-1.68	0.09
Power × Sex	-0.69	-1.85	0.06	-0.33	-0.38	0.70	-0.09	-0.18	0.85	1.75	3.27	<0.01^*^
HR × Sex	0.51	1.56	0.12	0.30	0.35	0.73	-0.23	-0.46	0.65	-2.01	-3.10	<0.01^*^
Power × [H^+^]	0.02	0.36	0.72	-0.23	-2.06	0.04^*^	-0.15	-2.40	0.02^*^	-0.35	-5.57	<0.01^*^

**Random effects**	***Intercept***		***Residual***	***Intercept***		***Residual***	***Intercept***		***Residual***	***Intercept***		***Residual***

Subject intercept	0.007		0.860	0.181		1.678	0.052		1.100	0.282		0.624
**Model *R***^2^	0.93			0.74			0.90			0.81		


*^∗^Indicates *p* < 0.05.*

#### Anxiety of Breathing

Results of the full model fit demonstrated a positive effect of [H^+^], and a negative interaction between power and [H^+^] (Table 1). The reduced model also demonstrated significant, positive effects of power and [H^+^], with a negative interaction between power and [H^+^], and a model *R*^2^-value of 0.74 (Figure 4 and Supplementary Table 3).

#### Leg Discomfort

Results of the full model fit for leg discomfort also demonstrated a positive effect of [H^+^], and a negative interaction between power and [H^+^] (Table 1). The reduced model of leg discomfort also demonstrated significant, positive effects of power, heart rate and [H^+^], with a negative interaction between power and [H^+^], and a model *R*^2^-value of 0.90 (Figure 4 and Supplementary Table 3).

#### Anxiety of Leg Discomfort

Results of the full model fit demonstrated positive effects of heart rate, [H^+^], [Lactate^-^], and an interaction between power and sex (male) (Table 1). Negative effects were shown for [Glucose], an interaction between heart rate and sex, and an interaction between power and [H^+^] (Table 1). The reduced model demonstrated significant, positive effects of heart rate, sex (male), [H^+^], [Lactate^-^], and an interaction between power and sex (male) (Figure 4 and Supplementary Table 3), with negative effects of power, [βHB^-^] and [Glucose], and negative interactions between heart rate and sex, and between power and [H^+^]. This reduced model had an *R*^2^-value of 0.81 (Figure 4).

## Discussion

In this study, we aimed to investigate the subcomponents of RPE and their driving physiological factors. We quantified subjective measures of breathlessness and leg discomfort as contributing components toward RPE, and observed no change in RPE during ketoacidosis despite widespread changes in peripheral exercising physiology. For the isolated perceptual components measured, breathlessness intensity was the only perception unaltered by ketoacidosis, and simultaneously the only perception that was not predicted by changes in blood pH. Whilst anxiety of breathing and intensity of leg discomfort contained pH (hydrogen ion concentration) as a humoral predictor, anxiety of leg discomfort was also associated with a number of other humoral signals, including the concentration of blood βHB ions.

The relationship between perceptions of exertion and physical work was first investigated by Borg and Dahlström in the 1950s (Borg and Dahlström, 1960). Within this early work, it was noted that power functions (with an exponent of approximately 1.6) best described the psychophysical relationship between perceptual changes and exercise intensity (Borg, 1982). These observations led Borg to conclude that RPE may encompass both more linear afferent factors, such as heart rate, and non-linear peripheral factors, such as lactate (exponent of approximately 2) (Borg, 1973, 1982). Therefore, Borg went on to develop the 15-point ‘Borg scale’ as a singular measure of perceived exertion (Borg, 1970, 1982, 1998), designed to increase linearly and reduce inter-subject variability by using carefully placed word anchors on the scale. In this manuscript, we aimed to decompose the RPE measure into perceptual components during incremental exercise, and assess how the underlying physiological signals contribute to these compartmentalized perceptions. Furthermore, we also incorporated measures of psychological affect (anxiety) toward these intensity perceptions. To model these perceptions and capture all signaling components in their native state, we used linear perception scales (0–10) and generalized linear mixed model regression, to account for inter-subject variability and non-normally distributed data (Faraway, 2017).

### Rating of Perceived Exertion

In extension of the seminal work by Borg and Dahlström (1960) and Borg (1962), modern models of RPE have theorized it to be more than simple modulation of afferent feedback (Ulmer, 1996; Hampson et al., 2001; Tucker, 2009). Subjective scores of RPE are thought to also encompass feedforward anticipation of exercise demands, allowing appropriate pacing strategies to be selected under a variety of physiological circumstances (Hampson et al., 2001; Noakes, 2004; Tucker, 2009). Furthermore, the more holistic measure of exercise ‘exertion’ is now also thought to be distinct from perception of exercising ‘effort’ (Abbiss et al., 2015). This idea addresses the breakdown of the relationship between RPE and exercise intensity under conditions of hypnosis (Morgan et al., 2008, 2011), or when key physiological signals are perturbed, such as heart rate via blocking agents (Ekblom and Golobarg, 1971), or in glycogen-deplete states (Baldwin et al., 2003; Noakes, 2004). Therefore, we created a null model of RPE encompassing power, heart rate, and sex (and their interactions), to account for linear and potential anticipatory effort perceptions with increasing power, as well as a measure of cardiac sympathetic drive via changes in heart rate, and any differences according to sex. Whilst this model demonstrated a tight fit to the RPE measures, positive, independent components of breathlessness, leg discomfort and the interaction between breathlessness and power were then shown to also independently contribute and improve this model, indicating each to be a significant component of RPE. Breaking down RPE into localized components may deepen our understanding of the independent contributions of afferent feedback from different bodily sensations, and thus equip us with better tools to explore how this may change across sporting modalities and/or contexts.

Whilst RPE had also originally been theorized to be influenced by psychological affect (Borg, 1998), previous work has also been unable to substantiate this relationship (Hardy and Rejeski, 1989). Therefore, it is possible that measuring RPE alone when investigating exercising perceptions may miss the influence of heavily valent affective emotions such as anxiety, which may also influence performance (Leupoldt von and Dahme, 2005; Banzett et al., 2008; Faull et al., 2016). Here we found no influence of breathlessness anxiety nor anxiety of leg discomfort toward RPE, although it must be noted that the relatively small sample size may hinder our ability to detect potentially small contributions from these variables. These preliminary data thus appear to indicate that perceptual anxiety does not positively contribute to subjective perceptions of RPE. However, as adapted linear scales were employed in this study (allowing for generalized linear models to be applied within the analysis) and the specific wording proposed by Borg to describe RPE (“how laborious it feels to work”; Borg, 1962) were not used in the description of the ratings to participants, care must be taken in the application of these results to other studies employing measures of RPE.

Lastly, RPE was not measurably altered during ketosis, despite widespread changes in physiology. These results further support a dominant component of anticipatory feedforward contributions to perceived exertion (Hampson et al., 2001; Tucker, 2009), even in the face of ketoacidosis. Therefore, whilst measures of RPE are able to garner enormous insight into man as a psychosomatic whole during exercise (Borg, 1982), these results highlights the need for additional, more interrogative measures of psychological affect to be simultaneously adopted. It would appear evolutionarily unlikely that either the presence of vital molecules such as βHB within the brain (Owen et al., 1967; Cahill and Owen, 1968; Cahill et al., 1970; Mikkelsen et al., 2015), or the concurrent changes in blood acidosis would not elicit any changes in perception of exercising exertion.

### Localized Perceptions of Exertion

Results of our RPE model revealed positive, independent contributions of breathlessness and leg discomfort perceptions toward overall exertion (RPE) during cycle ergometer exercise. Furthermore, these isolated perceptual intensity components were found to be driven by different physiological signals, where breathlessness contained only power and heart rate, while leg discomfort also contained pH as an integral predictor. In accordance with these modeling results, ketoacidosis did not affect breathlessness perception, while leg discomfort was increased compared to the control condition. Interestingly, leg discomfort was altered in a non-uniform fashion across exercising intensities with ketosis (Figure 2). It appears that the effect of pH on leg discomfort also depends on the intensity of the exercise, as demonstrated by the negative interaction effect between power and [H^+^]. Lower and moderate intensities of exercise were more susceptible to increased leg perception with acidosis, as this may create a peripheral mismatch when monitoring blood pH against what is expected for these exercise intensities (Paulus and Stein, 2006).

### Anxiety of Exercising Perceptions

The anxiety associated with interoceptive perception is an important, highly valent driver in the maintenance of homeostasis (Craig, 2003; Paulus and Stein, 2006). Contrary to intensity perceptions, anxiety of *both* breathlessness and leg discomfort were increased throughout ketoacidosis, and contained blood pH as a predictor of perception. Therefore, it is possible that anxiety perception measures are more broadly sensitive to potentially harmful peripheral changes in metabolism, and our homeostatic monitoring of these signals manifest more strongly within the affective perceptual domain. Furthermore, anxiety of leg discomfort appears to be a more complex measure that incorporates further humoral signals, including glucose and βHB. Both glucose and βHB cross the blood–brain barrier (Mikkelsen et al., 2015) and are important signals denoting metabolic stress. The combination of blood glucose and ketone ion concentrations represent two important components of cerebral fuel availability (Owen et al., 1967; Cahill and Owen, 1968; Cahill et al., 1970; Mikkelsen et al., 2015), and if exercise compromises that fuel it may heighten anxiety of leg discomfort. Therefore, perception of exercising limb anxiety may be a homeostatic warning signal whilst exercising under compromised metabolic conditions, such as during starvation. However, further modeling would be required to reproduce and thoroughly investigate these results in the future, incorporating larger datasets for more nuanced and generalisable statistical analyses.

## Conclusion

In this manuscript, we have conducted a preliminary investigation into the potential localized lung and leg perceptual components that contribute to RPE, and the importance of simultaneous affective anxiety perceptions. While the metabolic state of ketoacidosis does not appear to alter RPE, we reported concomitant increases in leg discomfort and affective perceptions of anxiety. In comparison, breathlessness appears to be unaffected by ketoacidosis. Finally, we have revealed that anxiety of leg discomfort may be a complex measure that incorporates humoral signals such as glucose and ketone concentrations, and thus potentially an evolutionarily important perception to help moderate exercise during times of metabolic stress. Whilst this data has yielded interesting preliminary modeling results of these perceptions, large scale datasets (with a more equal gender balance and a diverse study population) would be required for greater understanding and further statistical support regarding definitive sub-components of RPE and the physiological signal contributions to exercising perceptions.

## Data Availability

The datasets generated for this study are available on request to the corresponding author.

## Author Contributions

OF, DD, and PC collected the data for this study. PC and KC supervised the project. OF completed the data analysis and wrote the initial manuscript. All authors contributed intellectual ideas towards the final manuscript.

## Conflict of Interest Statement

The intellectual property and patents covering the uses of ketone bodies and esters are owned by BTG, Ltd., University of Oxford, the NIH and TdeltaS, Ltd. Should royalties ever accrue from these patents, KC and PC as named inventors may receive a share of royalties as determined by the terms of the respective institutions. KC is director of TdeltaS, a spin out company of the University of Oxford, to develop and commercialize products based on the ketone ester. OF was an employee of TdeltaS, Ltd. during the data collection and analysis for this manuscript, and DD is a current employee of TdeltaS, Ltd.
