# Supplementary material for: Beyond RPE: The Perception of Exercise Under Normal and Ketotic Conditions
Source: Front Physiol. 2019 Mar 19;10:229. doi: 10.3389/fphys.2019.00229 (PMC6433983; doi:10.3389/fphys.2019.00229)
Supplement: Supplementary file 1 [file Data_Sheet_1.pdf]

Supplementary Material

Beyond RPE: The perception of exercise under normal and ketotic conditions

Olivia K Faull[1,2], David J Dearlove[1], Kieran Clarke[1], Pete J Cox[1]

[1]Department of Physiology, Anatomy & Genetics, [2]Nuffield Department of Clinical Neurosciences University of Oxford, Oxford, UK

Corresponding author:

Dr Olivia Faull

Translational Neuromodeling Unit

Institute for Biomedical Engineering

University of Zurich and ETH Zurich

Email: faull@biomed.ee.ethz.ch

Phone: +41 (0)44 634 91 36

Supplementary materials and methods

Conversions for arterialised blood-gas corrections

To convert the measured pH and HCO_3^- into approximate arterial values, a 'buffer line' was drawn through the venous pH and HCO_3^- values (denoted as venpH and venHCO_3^- respectively) with a gradient of –(8.2 + 9.2 * [Hb]. The buffer line was then shifted to the left by a distance of 0.1 * (0.98 – SvO_2), where SvO_2 is the oxygen saturation in venous blood. This shift represents the increased saturation of haemoglobin in arterialised blood and, therefore, reduced buffering capacity. This line is given by the equation: [HCO_3^-] =-(8.2+9.2*Hb)*pH+venHCO_3^- +a*venpH-a*0.1*(0.98-SvO_2).

The intersection point between the second buffer line and the line given by the Henderson-Hasselbalch equation pH = pK + $\log_{10}$([HCO_3^-]/(α.P_{CO2})) in the Davenport diagram was sought by rearranging the equation: [HCO_3^-] = α.P_{CO2}..10^(pH-pK). The root-finding method fsolve in MATLAB was then applied to find the point where these two curves meet, which gives the arterial values for [HCO_3^-] and pH. This description is taken from Dearlove et al. (in submission).

Rating scales

Rating of perceived exertion	Breathlessness Intensity	Anxiety of Breathing	Leg Discomfort	Anxiety of leg discomfort
0 No exertion	0 No breathlessness	0 No breathing anxiety	0 No leg discomfort	0 No leg anxiety
1	1	1	1	1
2	2	2	2	2
3	3	3	3	3
4	4	4	4	4
5	5	5	5	5
6	6	6	6	6
7	7	7	7	7
8	8	8	8	8
9	9	9	9	9
10 Extremely hard Maximal exertion	10 Extremely hard Maximal breathlessness	10 Extremely hard Maximal breathing anxiety	10 Extremely hard Maximal leg discomfort	10 Extremely hard Maximal leg discomfort anxiety

Supplementary results

Supplementary Table 1. ANOVA results

Perception	Variable	DF	*F* value	*P* value
**RPE**	Power	11	62.41	< 0.01*
	Ketone ester	1	0.09	0.77
	Interaction	11	0.57	0.85
**Breathlessness**	Power	11	50.70	< 0.01*
	Ketone ester	1	1.11	0.29
	Interaction	11	0.42	0.95
**Anxiety of breathing**	Power	11	12.55	< 0.01*
	Ketone ester	1	4.75	0.03*
	Interaction	11	0.71	0.73
**Leg discomfort**	Power	11	44.47	< 0.01*
	Ketone ester	1	12.91	< 0.01*
	Interaction	11	2.11	0.02*
**Anxiety of leg discomfort**	Power	11	10.04	< 0.01*
	Ketone ester	1	8.33	< 0.01*
	Interaction	11	0.63	0.80

Variables included in mixed effects regression models

RPE models:

- Power: Included to account for any linear relationship between RPE and increasing power.
- Heart rate: Included to account for any relationship between RPE and heart rate, as a measure of central cardiac sympathetic response to exercise intensity.
- Sex: Included to account for any differences in RPE between males and females.
- Breathlessness: Included to account for any independent effects of perceptions of breathlessness towards RPE scores.
- Leg discomfort: Included to account for any independent effects of perceptions of leg discomfort towards RPE scores.
- Anxiety of breathing: Included to account for any independent effects of perceptions of breathlessness anxiety towards RPE scores.
- Anxiety of leg discomfort: Included to account for any independent effects of perceptions of anxiety of leg discomfort towards RPE scores.
- Interaction terms (included following all singular fixed-effect terms): These allow for the influence of one independent variable on RPE to change according the magnitude of another variable. These included interactions between power and heart rate (to account for possible factors such non-linearity in heart rate increases and heart rate plateaus despite added sympathetic during heavy exercise), power and sex (to account for possible power-dependent influences of sex), and heart rate and sex (to account for possible heart rate-dependent influences of sex) for the null model, and additional interactions between power and leg discomfort and power and breathlessness (to account for non-linear changes in these variables as power increases) for the initial formulation of the hypothesis model.
- Random subject intercept effect: Allows each subject to be modelled with their own regression line with a different intercept.

Psychological component global models:

- Power: Included to account for any linear relationship between the modelled perception score and increasing power.
- Heart rate: Included to account for any relationship between the modelled perception score and heart rate, as a measure of central cardiac sympathetic response to exercise intensity.
- Sex: Included to account for any differences in the modelled perception score between males and females.
- [H^+]: Included to account for the effect of circulating hydrogen ion concentration on the modelled perception score.
- [$Lactate^-$]: Included to account for the effect of circulating lactate ion concentration on the modelled perception score.
- Ventilation: Included to account for the effect of increasing ventilation on the modelled breathing-related perception scores (included for breathlessness and breathlessness anxiety modelling only).
- [βHB^-]: Included to account for the effect of circulating β-hydroxybutyrate ion concentration on the modelled perception score.
- [Glucose]: Included to account for the effect of circulating glucose concentration on the modelled perception score.
- Interaction terms (included following all singular fixed-effect terms): These allow for the influence of one independent variable on the modelled perception score to change according the magnitude of another variable. These included interactions between power and heart rate (to account for possible factors such non-linearity in heart rate increases and heart rate plateaus despite added sympathetic drive during heavy exercise), power and sex (to account for possible power-dependent influences of sex), heart rate and sex (to account for possible heart rate-dependent influences of sex), and power and [H^+] (to account for any power-dependent influences of the circulating hydrogen ion concentration).
- Random subject intercept effect: Allows each subject to be modelled with their own regression line with a different intercept.

Mixed effects regression model parameters

Supplementary Table 2. RPE null and full model parameters

	**Variable**	***Coeff***	***T* value**	***P* value**	**R^2**
**Null RPE model**	Intercept	2.68	17.49	< 0.01*	-
	Power	1.00	4.13	< 0.01*	0.13
	Heart rate	-0.01	-0.05	0.96	< 0.01
	Sex (male)	-0.46	-4.07	< 0.01*	0.29
	Power x heart rate	-0.15	-3.21	< 0.01*	0.24
	Power x sex (male)	-0.55	-2.27	0.02*	0.04
	Heart rate x sex (m)	0.55	2.34	0.02*	0.06
		Intercept		***Residual***	
(Random effect)	Subject intercept	0.006		0.728	
**Final RPE model**	Intercept	1.63	29.98	< 0.01*	-
	Power	0.33	4.24	< 0.01*	0.22
	Sex (male)	-0.26	-3.71	< 0.01*	0.29
	Leg discomfort	0.12	3.00	< 0.01*	0.08
	Breathlessness	0.38	7.53	< 0.01*	0.31
	Power x sex	0.17	2.79	< 0.01*	0.13
	Power x breathlessness	-0.24	-10.72	< 0.01*	0.58
		Intercept		***Residual***	
(Random effect)	Subject intercept	0.003		0.392	

Supplementary Table 3. Final compartmentalised model parameter results

	Variable	*Coeff*	*T* value	*P* value	R^2
**Breathlessness**	Intercept	1.29	15.50	< 0.01	-
	Power	0.31	4.13	< 0.01	0.14
	Heart rate	0.61	6.47	< 0.01	0.39
	Sex (male)	-0.30	-3.39	< 0.01	0.19
		Intercept		***Residual***	
(Random effect)	Subject intercept	0.008		1.028	
**Anxiety of breathing**	Intercept	0.76	4.83	< 0.01	-
	Power	0.90	8.41	< 0.01	0.53
	$[H^+]$	0.41	4.18	< 0.01	0.16
	Power x $[H^+]$	-0.33	-3.56	< 0.01	0.19
		Intercept		***Residual***	
(Random effect)	Subject intercept	0.305		1.771	
**Leg discomfort**	Intercept	1.15	13.71	< 0.01	-
	Power	0.52	3.85	< 0.01	0.20
	Heart rate	0.37	2.46	0.01	0.11
	$[H^+]$	0.23	3.73	< 0.01	0.18
	Power x $[H^+]$	-0.20	-3.59	< 0.01	0.22
		Intercept		***Residual***	
(Random effect)	Subject intercept	0.047		1.186	
**Anxiety of leg discomfort**	Intercept	0.23	0.41	0.68	-
	Power	-0.69	-1.46	0.15	0.01
	Heart rate	1.95	3.38	< 0.01	0.08
	Sex (male)	0.79	1.36	0.17	0.09
	$[H^+]$	0.30	4.64	< 0.01	0.06
	$[Lac^-]$	0.15	3.02	< 0.01	0.01
	$[\beta HB^-]$	-0.05	-1.97	0.05	0.01
	[Glucose]	-0.05	-2.02	0.04	< 0.01
	Heart rate x sex (m)	-1.71	-2.76	0.01	0.06
	Power x sex (m)	1.33	2.77	0.01	0.02
	Power x $[H^+]$	-0.39	-6.52	< 0.01	0.14
		Intercept		***Residual***	
(Random effect)	Subject intercept	0.286		0.644	

Mixed effects regression model diagnostics

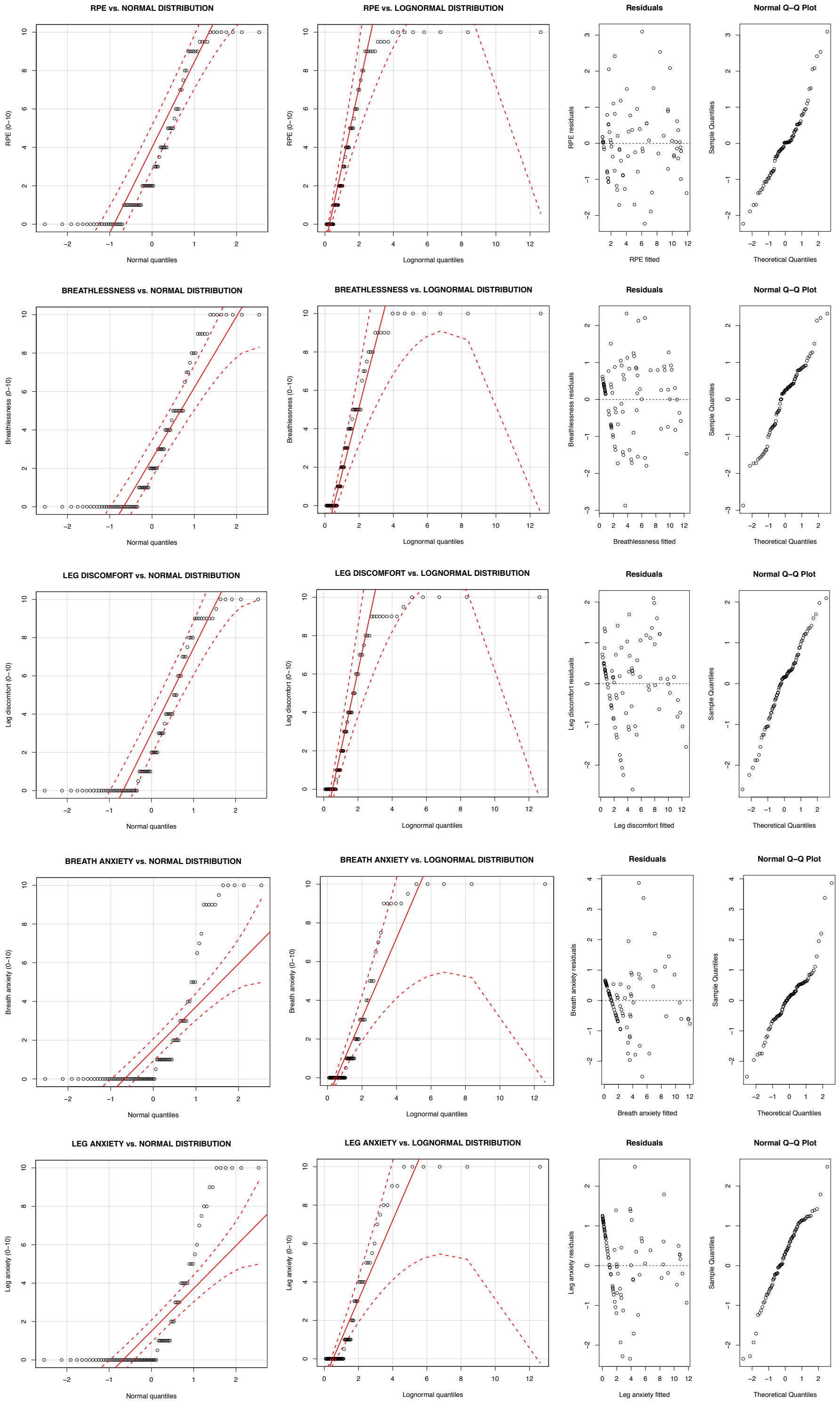

Supplementary Figure 1. Data and residual diagnostics for generalised mixed effects models. For each model, the distribution of the dependent variable more closely matches a log-normal than a normal distribution (left two plots of each row). Following model fitting, residuals display relative homoskedasticity and normal distributions.
